# MRAP2 regulates energy homeostasis by promoting primary cilia localization of MC4R

**DOI:** 10.1172/jci.insight.155900

**Published:** 2023-01-24

**Authors:** Adelaide Bernard, Irene Ojeda Naharros, Xinyu Yue, Francois Mifsud, Abbey Blake, Florence Bourgain-Guglielmetti, Jordi Ciprin, Sumei Zhang, Erin McDaid, Kellan Kim, Maxence V. Nachury, Jeremy F. Reiter, Christian Vaisse

**Affiliations:** 1 Department of Medicine and The Diabetes Center,; 2 Department of Ophthalmology, and; 3 Department of Biochemistry and Biophysics, Cardiovascular Research Institute, UCSF, San Francisco, California, USA.; 4 Chan Zuckerberg Biohub, San Francisco, California, USA.

**Keywords:** Endocrinology, Metabolism, Melanocortin, Neuroendocrine regulation, Obesity

## Abstract

The G protein–coupled receptor melanocortin-4 receptor (MC4R) and its associated protein melanocortin receptor–associated protein 2 (MRAP2) are essential for the regulation of food intake and body weight in humans. MC4R localizes and functions at the neuronal primary cilium, a microtubule-based organelle that senses and relays extracellular signals. Here, we demonstrate that MRAP2 is critical for the weight-regulating function of MC4R neurons and the ciliary localization of MC4R. More generally, our study also reveals that GPCR localization to primary cilia can require specific accessory proteins that may not be present in heterologous cell culture systems. Our findings further demonstrate that targeting of MC4R to neuronal primary cilia is essential for the control of long-term energy homeostasis and suggest that genetic disruption of MC4R ciliary localization may frequently underlie inherited forms of obesity.

## Graphical abstract

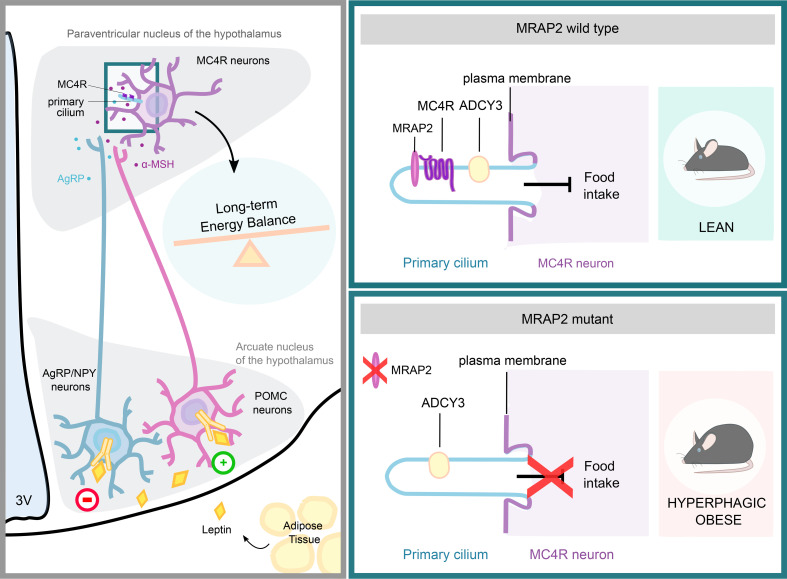

## Introduction

The regulation of food intake and energy expenditure is dependent on the genetic, molecular, and cellular integrity of the central melanocortin system, a network of hypothalamic neurons that integrate peripheral information about energy status and regulate long-term energy homeostasis, thereby preventing obesity (1). This system comprises the neuropeptides α-MSH and AGRP, produced by independent populations of neurons in the arcuate nucleus, which are sensitive to the adipocyte-secreted hormone leptin, as well as the receptor for these neuropeptides, the melanocortin-4 receptor (MC4R). MC4R, 1 of 5 members of the Gαs-coupled melanocortin receptor family (2), is found in multiple brain regions, but its expression in the paraventricular nucleus (PVN) of the hypothalamus is both necessary and sufficient for the regulation of food intake and body weight (3, 4). Underscoring the essential role of this receptor in the maintenance of energy homeostasis, heterozygous loss-of-function mutations in *MC4R* are the most common cause of monogenic obesity in humans (5–7), and the *MC4R* locus displays the second strongest association with obesity among the common variants influencing BMI (8–10). *Mc4r^+/–^* and *Mc4r^–/–^* mice recapitulate the obesity phenotypes observed in humans (11). The central importance of MC4R in energy homeostasis has made it a major target for the pharmacotherapy of obesity. However, little is known about the molecular and cellular pathways underlying the maintenance of long-term energy homeostasis by MC4R-expressing neurons.

Recently, we reported that MC4R localizes and functions at the neuronal primary cilium (12, 13), a cellular organelle that projects from the surface of most mammalian cell types and functions as an antenna to sense extracellular signals (14). Inherited mutations that disrupt ciliary structure or function cause ciliopathies (15–17), disorders characterized by pleiotropic clinical features that can include hyperphagia and severe obesity (18), such as in Alström or in Bardet-Biedl syndrome (BBS). In adult mice, genetic ablation of neuronal primary cilia also causes obesity (19).

MC4R physically interacts with melanocortin receptor–associated protein 2 (MRAP2), a member of the MRAP family composed of single-pass transmembrane proteins that interact with melanocortin receptors (20) and other GPCRs (21, 22). In nonciliated heterologous systems, MRAP2 binds to MC4R and increases ligand sensitivity, as well as MC4R-mediated generation of cAMP (23–25). In humans, MRAP2 variants were found in patients with obesity (23, 26–28). *Mrap2^–/–^* mice develop severe obesity, although they lack the early-onset hyperphagia of *Mc4r*^–/–^ mice (23), which brings into question the extent to which MRAP2 qualitatively and quantitatively interacts with MC4R in vivo. Indeed, MRAP2 has also been suggested to interact with other GPCRs such as the ghrelin receptor and the prokineticin receptor (21, 22).

Here, we find that the central mechanism of MRAP2-associated obesity is the critical role for MRAP2 in targeting MC4R to cilia.

## Results

### MC4R neurons require MRAP2 to regulate energy homeostasis.

Germinal deletion of MRAP2, in *Mrap2*^–/–^ mice, leads to obesity, although to a lesser extent than that observed in *Mc4r*^–/–^ mice (23). To determine whether MRAP2 is essential for MC4R neurons to control food intake and body weight, we deleted MRAP2 from MC4R-expressing cells. We obtained mice bearing an *Mrap2*-KO–first (“tm1a”) allele and confirmed that, as previously reported (20, 23, 29), homozygous mutant mice (hereafter referred to as *Mrap2^–/–^*) (Supplemental Figure 1A; supplemental material available online with this article; https://doi.org/10.1172/jci.insight.155900DS1) were obese and hyperphagic at 12 weeks of age but did not differ from their WT littermates at 4 weeks of age (Supplemental Figure 1, B and C). From these mice, we generated an *Mrap2*-floxed allele (“tm1c” allele, hereafter referred to as *Mrap2^fl^*) (Supplemental Figure 1A). *Mrap2^fl/fl^* mice weighed the same as their WT littermates (Supplemental Figure 1D).

**Figure 1:**
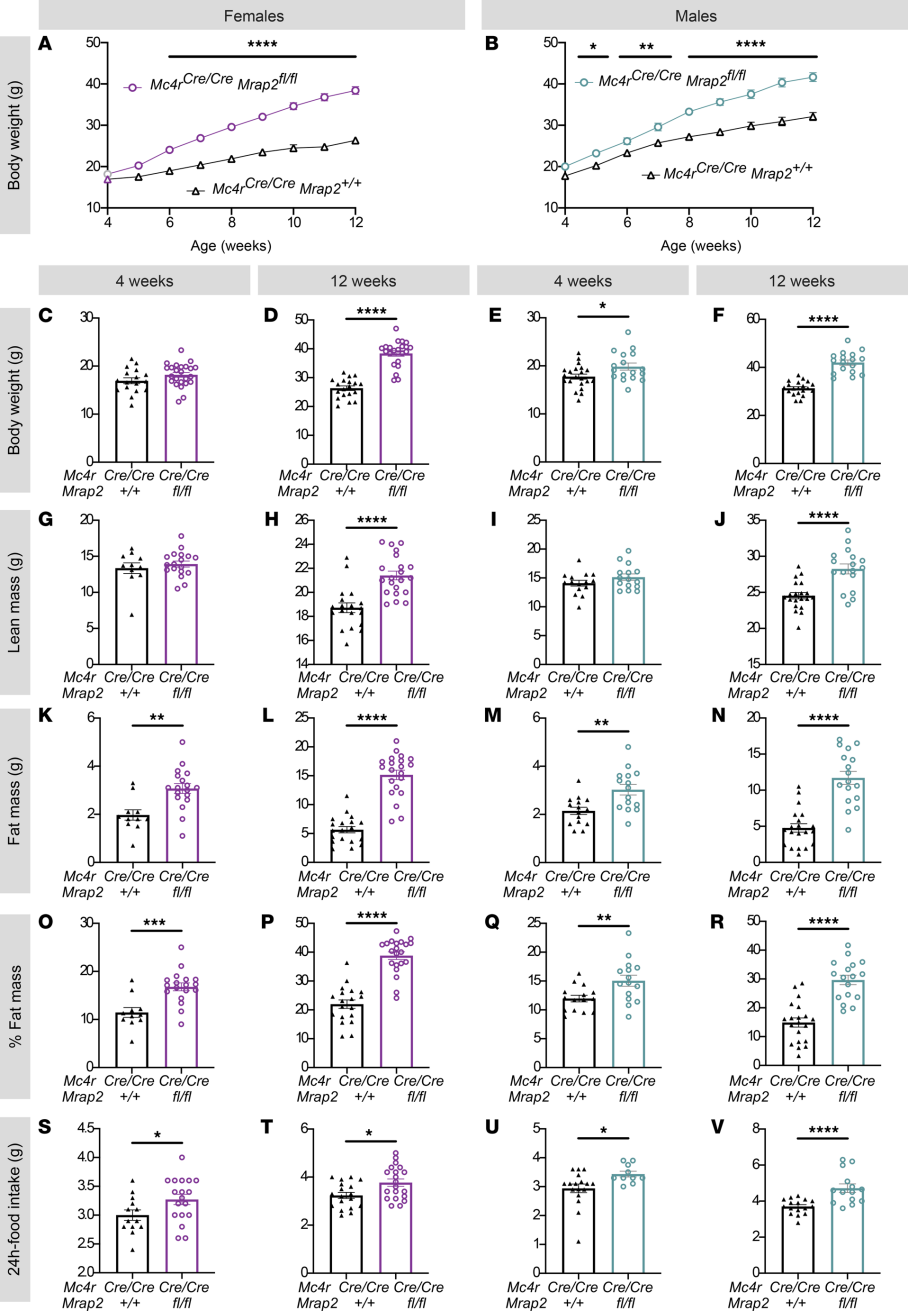
MRAP2 functions in MC4R-expressing cells to regulate food intake and restrain body weight. (**A** and **B**) Body weight curve of *Mc4r^t2aCre/t2aCre^* *Mrap2^fl/fl^* versus *Mc4r^t2aCre/t2aCre^ Mrap2^+/+^* female (**A**) and male (**B**) littermates. (**C**–**R**) Respective body composition at 4 and 12 weeks of *Mc4r^t2aCre/t2aCre^* *Mrap2^fl/fl^* versus *Mc4r^t2aCre/t2aCre^ Mrap2^+/+^* females (body weight [**C** and **D**], lean mass [**G** and **H**], fat mass [**K** and **L**], percent fat mass [**O** and **P**]) and males (body weight [**E** and **F**], lean mass [**I** and **J**], fat mass [**M** and **N**], percent fat mass [**Q** and **R**]). (**S**–**V**) Twenty-four–hour food intake at 4 and 12 weeks of *Mc4r^t2aCre/t2aCre^* *Mrap2^fl/fl^* versus *Mc4r^t2aCre/t2aCre^* *Mrap2^+/+^* females (**S** and **T**) and males (**U** and **V**). *n* = 11–24 mice per group were used, individual values are displayed, and exact number of mice per panel is detailed in the methods. Data are represented as mean ± SEM; *P < 0.05, **P < 0.01, ***P < 0.001, ****P < 0.0001 by 2-tailed Student’s unpaired *t* test (column analysis; **C**–**V**), mixed-effects model (REML), and Sidak’s multiple-comparison tests (weight curves; **A** and **B**). The exact number of mice displayed in each panel is as follows, with WT indicating *Mc4r^t2aCre/t2aCre^ Mrap2^+/+^* and mut indicating *Mc4r^t2aCre/t2aCre^* *Mrap2^fl/fl^*. Body weight: *n* = 19 WT females (F); 24 mut F; 20 WT males (M); 17mut M. EchoMRI at 4 weeks old: *n* = 13 WT F; 18 mut F; 17 WT M; 11 mut M. EchoMRI at 12 weeks old: *n* = 18 WT F; 20 mut F; 17 WT M; 14 mut M. Food intake at 4 weeks old: *n* = 13 WT F; 18 mut F; 17 WT M; 11 mut M. Food intake at 12 weeks old: *n* = 18 WT F; 20 mut F; 17 WT M; 14 mut M.

To specifically delete MRAP2 in MC4R-expressing neurons throughout the brain, we generated *Mc4r*-*t2a-Cre* (13) *Mrap2^fl/fl^* mice (hereafter referred to as *Mc4r^t2aCre/t2aCre^* *Mrap2^fl/fl^*). *Mc4r^t2aCre/t2aCre^ Mrap2^fl/fl^* mice fed ad libitum regular chow developed early-onset obesity with significantly higher body weight (Figure 1, A–F) and fat mass (Figure 1, K–R), associated with hyperphagia (Figure 1, S–V) compared with their *Mc4r^t2aCre/t2aCre^ Mrap2^+/+^* littermates.

This phenotype was apparent at 4 weeks of age (Figure 1, C, E, K, M, O, Q, S, and U) and was present both in females and in males (Figure 1). No differences in energy expenditure (EE) or activity were observed between groups, both in females and in males (Supplemental Figure 2). Together, these data demonstrate that MRAP2 is essential in MC4R-expressing neurons to regulate food intake and body weight.

### MRAP2 promotes MC4R targeting to the primary cilium in heterologous cells.

The interaction between MRAP2 and MC4R, as well as the cellular localization of MRAP2, have been previously studied in nonciliated cells (23–26, 30). Since MC4R localizes to the primary cilium, and MRAP2 has been reported to interact with MC4R in vitro (23), we tested whether MRAP2 colocalizes with MC4R at the primary cilium following transient transfection in murine ciliated inner medullary collecting duct 3 (IMCD3) cells.

**Figure 2:**
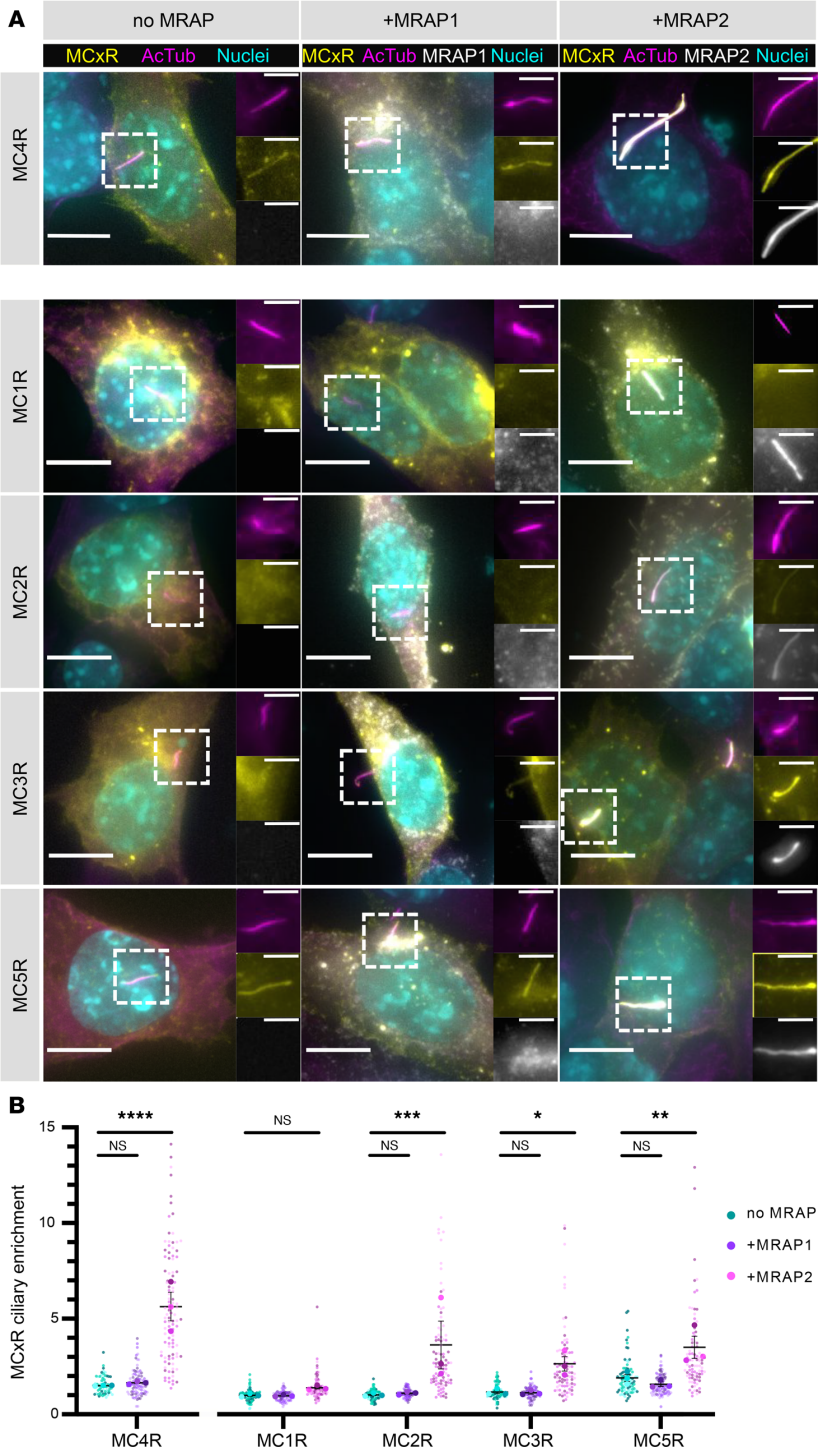
MRAP2 localizes MC4R to the IMCD3 primary cilium. (**A**) Representative widefield micrographs of IMCD3 cells transiently transfected with GFP-tagged melanocortin receptors alone (left), or cotransfected with MRAP1-FLAG (center) or MRAP2-FLAG (right). Cells are stained for cilia (AcTub, magenta), GFP-tagged melanocortin receptors (yellow), MRAP1- or MRAP2-FLAG (white), and nuclei (Hoechst 33342, cyan). MC4R-GFP and MRAP2-FLAG colocalize at the primary cilium (top panel). Scale bars: 5 μm (low-magnification images) and 2 μm (inserts). (**B**) Melanocortin receptor enrichment at the cilium when transfected without MRAP (blue), with MRAP1 (purple), or with MRAP2 (magenta). MRAP2 expression increases ciliary localization of MC2R, MC3R, MC4R, and MC5R but not MC1R. MRAP1 coexpression has no effect on ciliary localization. *n* = 20–40 ciliated cells per condition were imaged and analyzed. Ciliary and cell body intensity of melanocortin receptors and MRAPs was measured using Fiji. Enrichment at the cilium is expressed as: (integrated density at the cilium)/(integrated density in the cell body). Enrichment > 1 indicates higher localization of GFP-tagged melanocortin receptor or MRAP at the primary cilium than at the cell body. Data are from 3 different replicates and are represented as a superplot, with their mean and SEM. *P < 0.05, **P < 0.01, ***P < 0.001, ****P < 0.0001 using ordinary 2-way ANOVA, with Tukey’s multiple-comparison test performed on replicate averages.

We found that both MRAP2 and MC4R colocalized with the ciliary shaft labeled by acetylated tubulin (AcTub; Supplemental Figure 3B) and that ciliary localization of MC4R was strongly increased when MRAP2 and MC4R were cotransfected (Figure 2A, top panel). We then determined whether MRAP2 localization is a common feature of members of the MRAP family and found that the MRAP1, a paralog of MRAP2 and an essential accessory factor for the functional expression of the MC2R/ACTH receptor, did not localize to the primary cilium (Figure 2, A and B, and Supplemental Figure 3, A and B).

We further tested to what extent MRAP2 promotes MC4R ciliary localization and found that MRAP2 increased MC4R enrichment at the primary cilium, whereas MRAP1 had no effect (Figure 2, A and B, top panel). Finally, we assessed the specificity of the interaction between MRAP2 and MC4R by systematically testing the ciliary enrichment of all 5 melanocortin receptor family members in the presence or absence of MRAPs (*n* = 30; Figure 2B and Supplemental Figure 3C). Although MRAP2 promoted the ciliary localization of other melanocortin receptors, the greatest effect was observed on MC4R (Figure 2B and Supplemental Figure 3C). MRAP1 did not affect ciliary enrichment of any of the receptors (Figure 2B).

Thus, MRAP2 specifically promotes MC4R localization to the primary cilia in heterologous cells.

### MRAP2 colocalizes with MC4R at the primary cilium of hypothalamic neurons in vivo.

**Figure 3:**
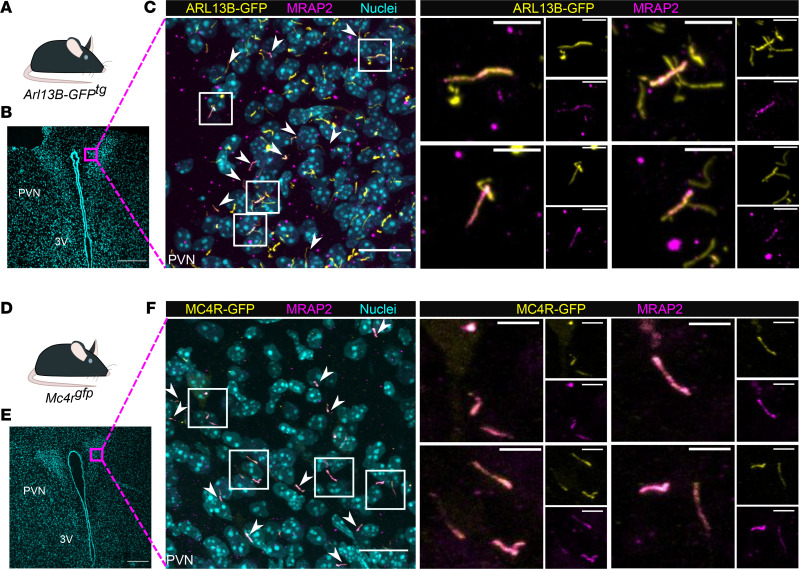
MRAP2 colocalizes with MC4R at the primary cilia in vivo. (**A**–**C**) MRAP2 localizes to primary cilia in vivo. (**A**) Mice expressing a transgene encoding a ciliary GFP (Arl13-GFP) were used in this experiment. (**B**) Representative low-magnification image of the PVN (representative of *n* = 3) and nuclei (Hoescht, cyan). Magenta square indicates higher-magnification image, depicted in **C**. Scale bar: 200 μm. (**C**) Immunofluorescence image of cilia (*Arl13b-GFP^tg^*, yellow), MRAP2 (magenta), and nuclei (Hoechst, cyan) in the mouse PVN, showing that MRAP2 localizes to primary cilia (arrows). (**D**–**F**) MRAP2 colocalizes with MC4R in vivo (arrows and boxes). (**D**) Mouse line expressing a GFP tag in frame at the C-terminus of the endogenous *Mc4r* locus. (**E**) Representative low-magnification image of the PVN (representative of *n* = 3) and nuclei (Hoescht, cyan). Magenta square indicates higher magnification image, depicted in **F**. Scale bar: 200 μm. (**F**) Immunofluorescence image of MC4R-GFP (yellow), MRAP2 (Magenta). and nuclei (Hoechst, cyan) in the mouse PVN. Indicated boxed regions are shown to the right at higher magnification. Scale bars: 20 μm (low-powered images) and 5 μm (high-powered images). Brains were collected from P6 mice. 3V, third ventricle; PVN, paraventricular nucleus of the hypothalamus. Arrowheads indicate cilia expressing both MRAP2 and arl13-GFP (**A**–**C**) or MRAP2 and MC4R (**D**–**F**).

MRAP2 subcellular localization was assessed by immunofluorescence, using a commercial antibody, the specificity of which was initially confirmed in vitro in transfected cells (Supplemental Figure 4A) and in vivo compared with MRAP2-KO sections (Supplemental Figure 4B). We first examined the subcellular localization of MRAP2 in a transgenic reporter mouse in which all primary cilia are labeled (*Arl13b-GFP^tg^*) (31). In these mice, MRAP2 was found to colocalize with some but not all primary cilia in the PVN of the hypothalamus (Figure 3, A–C).

To further determine whether MRAP2 colocalizes with MC4R at primary cilia, we used a mouse line in which GFP was fused to the C-terminus of MC4R at the endogenous locus (12) (hereafter referred to as *Mc4r^gfp^*), allowing for the assessment of the subcellular localization of the endogenous MC4R. In the PVN of *Mc4r^gfp^* mice, most MC4R localized at primary cilia (Supplemental Figure 5), and remarkably, MRAP2 localized exclusively to the cilium of these cells (Figure 3, D–F).

Since MC4R is widely expressed throughout the brain, we sought to determine whether MC4R colocalizes with MRAP2 at the cilium in other brain regions. We found that, although MRAP2 expression is broader than MC4R’s, MC4R always colocalizes with MRAP2 at the primary cilium in other nuclei where MC4R is expressed (Supplemental Figure 6).

### MRAP2 is required for MC4R localization to primary cilia in vivo.

Since MRAP2 enhances MC4R localization at primary cilia in vitro, and MC4R colocalizes with MRAP2 at primary cilia in vivo, we tested whether loss of MRAP2 compromises MC4R localization at primary cilia in vivo. We generated *Mrap2*^+/+^ *Mc4r^gfp^* and *Mrap2*^–/–^ *Mc4r^gfp^* mice to compare the ciliary localization of MC4R in the presence and absence of MRAP2.

**Figure 4:**
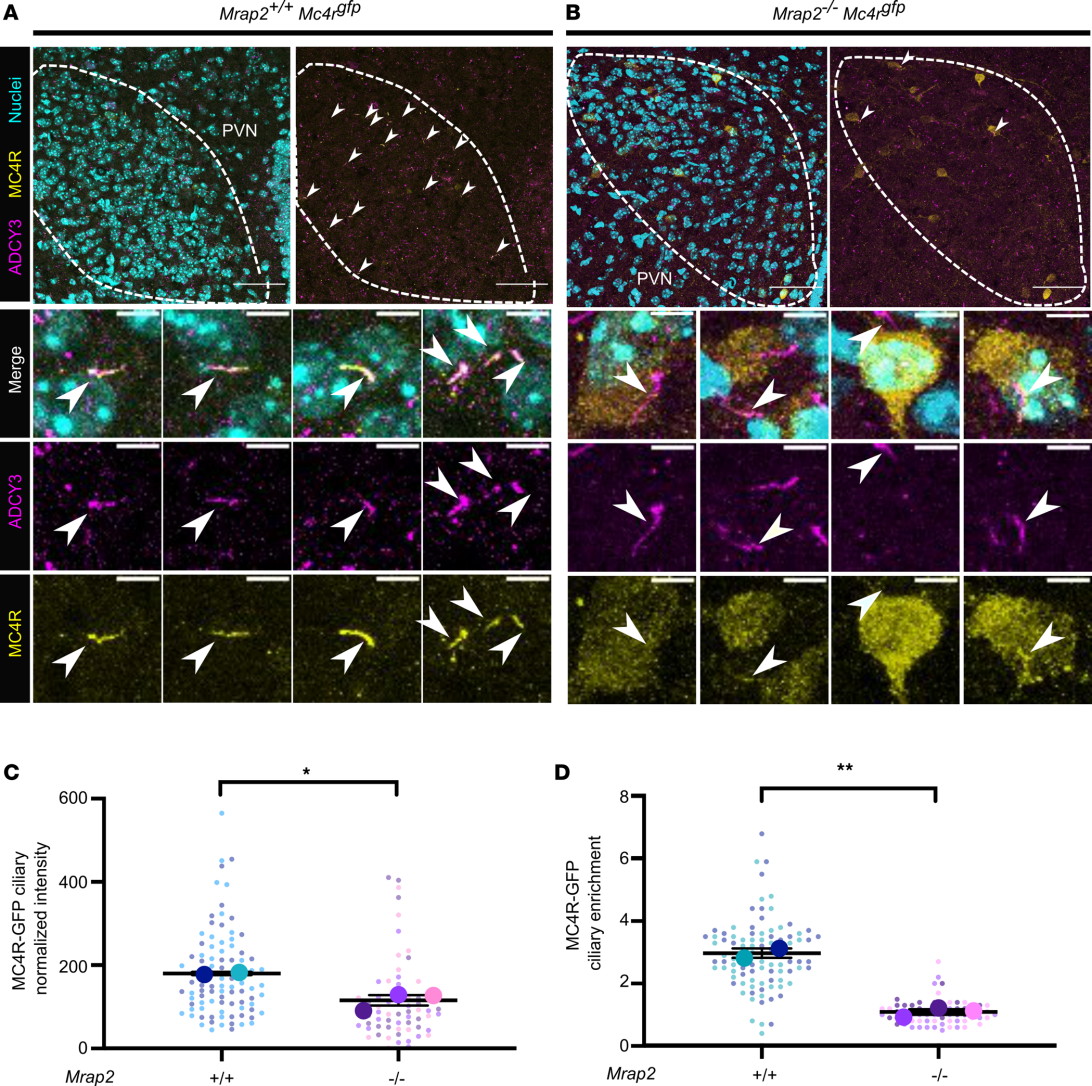
MRAP2 is required for MC4R localization to the primary cilia. (**A** and **B**) Immunofluorescence images of MC4R-GFP (yellow), cilia (ADCY3, magenta) and nuclei (Hoechst, cyan). Top panels: dashed lines delineate the PVNs. Scale bar: 50 μm. Bottom panels: High-magnification images. Scale: 5 μm. Arrowheads indicate MC4R-GFP^+^ cilia. (**C**) Quantitation of MC4R-GFP fluorescence intensity at the primary cilium (in arbitrary units). MC4R ciliary localization is decreased in the absence of MRAP2. (**D**) Quantitation of MC4R enrichment at the primary cilium as: (integrated density at the cilium)/(integrated density in an equal area of the cell body). Enrichment > 1 indicates higher localization of MC4R-GFP at the primary cilium than at the cell body. MC4R localization was quantified from 2 PVNs per P6 *Mc4r^gfp^* *Mrap2*^+/+^ (*n* = 2) and *Mc4r^gfp^* *Mrap2*^–/–^ (*n* = 3) mice; 60–90 cilia we quantified per mouse. Data are represented as superplots displaying individual cilia intensity and enrichment values (small dots), and 2-tailed Student’s *t* tests were performed on the averages per mouse (big dots), represented as mean ± SEM; *P < 0.05, **P < 0.01.

In the PVN of *Mrap2*^+/+^ *Mc4r^gfp^* mice, MC4R mainly localized to cilia (Figure 4A). Remarkably, in *Mrap2*^–/–^ *Mc4r^gfp^* mice, MC4R was rarely found at primary cilia and, in some cells, was detectable in the neuronal cell bodies (Figure 4B). Of note, we confirmed that no MRAP2 staining was detected in *Mrap2*^–/–^ *Mc4r^gfp^* mice — in particular, in the few cells in which MC4R localized to primary cilia — further confirming the specificity of the anti-MRAP2 antibody (Supplemental Figure 4B).

The normalized intensity of MC4R at cilia (defined by ADCY3 immunostaining) was decreased in *Mrap2* mutants compared with WT (Figure 4C), and MC4R was no longer enriched in cilia in the absence of MRAP2 (Figure 4D). Thus, MRAP2 is necessary for MC4R enrichment at primary cilia both in vitro and in vivo.

**Figure 5:**
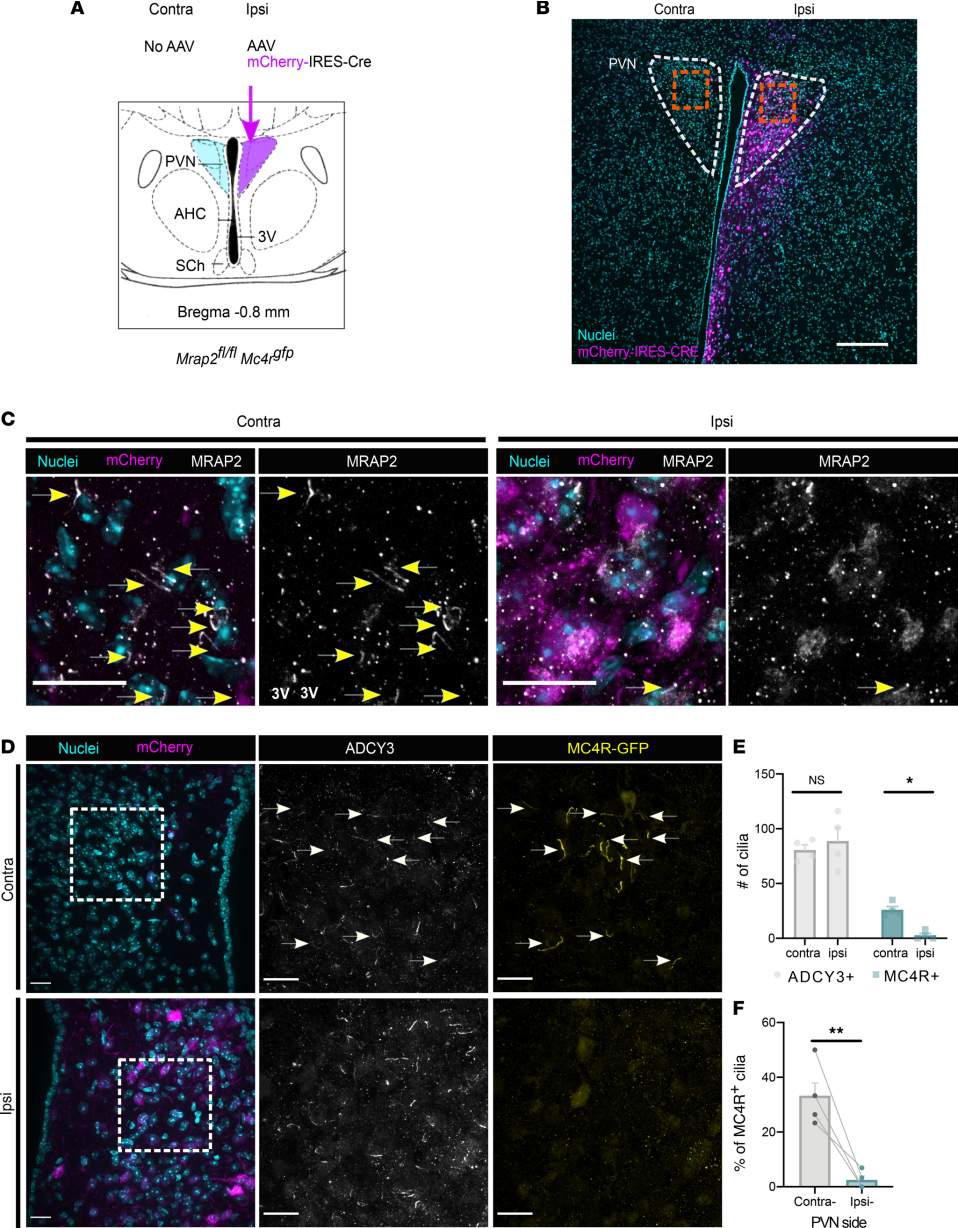
MRAP2 is required in the adult PVN for MC4R ciliary localization. (**A**) Experimental design. *Mrap2^fl/fl^ Mc4r^gfp^* mice were injected unilaterally with AAV encoding mCherry-IRES-Cre (*n* = 4, 8-week-old females) and analyzed 3 weeks following injection. (**B**) Representative low-magnification image of PVN, mCherry (magenta), and nuclei (Hoescht, cyan). Orange squares indicate higher-magnification images depicted in **C**. Scale bar: 200 μm. (**C**) Higher-magnification images of inserts from **B** (orange squares). Immunofluorescence staining of MRAP2 (white) of the control contralateral and experimental, ipsilateral PVN with mCherry (magenta), and nuclei (Hoescht, cyan). Arrows indicate MRAP2^+^ cilia, absent from mCherry-expressing cells. Scale bar: 50 μm. (**D**) Immunofluorescence staining of control, contralateral PVN, and experimental, ipsilateral PVN for, on the left, mCherry (magenta) and nuclei (Hoescht, cyan); in the center, ADCY3 (white); and, on the right, MC4R-GFP (yellow). White squares indicate regions imaged for higher-magnification images in the middle and right. ADCY3 is not altered by loss of MRAP2, while MC4R localization to neuronal primary cilia is abrogated by loss of MRAP2. Arrows indicate MC4R^+^ cilia. Scale bars: 20 μm. (**E**) Quantification of the total number of cilia (ADCY3^+^) and MC4R^+^ cilia per image in the control, contralateral PVN, and experimental, ipsilateral PVN. (**F**) Quantification of the percentage of MC4R^+^ cilia remaining in the ipsi-AAV–injected side compared with the contralateral side. Data are represented as mean ± SEM; *P < 0.05, **P < 0.01 using 2 way ANOVA with Sidak’s multiple-comparison test (**E**) and 2-tailed Student’s *t* test (**F**).

Lack of MC4R localization to primary cilia in *Mrap2*^–/–^ *Mc4r^gfp^* mice could be secondary, resulting from developmental effects. We therefore determined whether acute deletion of *Mrap2* in the PVN of adult mice would affect MC4R ciliary localization. We injected *Mrap2^fl/fl^ Mc4r^gfp^* mice unilaterally with an adeno associated virus (AAV) encoding mCherry-IRES-Cre. *Mrap2* was deleted from the infected PVN by Cre-mediated recombination, and the contralateral uninfected PVN served as an internal control (Figure 5, A and B). The brains were harvested 3 weeks following the AAV injections and were analyzed. While MRAP2 localized to cilia in the contralateral control PVN, MRAP2 staining was not detected in the mCherry-Cre–injected side, confirming deletion of the gene (*n* = 4; Figure 5, B and C). Cilia, identified by ADCY3 staining, were present in the infected PVN (Figure 5D), confirming that MRAP2 is dispensable for PVN primary cilia maintenance. Remarkably, MC4R localized to cilia in control PVNs but not in the PVNs from which MRAP2 had been removed (Figure 5, D–F), confirming that MRAP2 is essential for MC4R ciliary localization.

To further assess the specificity of MRAP2 in mediating the localization of MC4R to primary cilia, we tested whether MRAP2 contributes to the ciliary localization of other GPCRs. Specifically, we assessed the localization of the somatostatin receptor 3 (SSTR3) in the presence or absence of MRAP2. SSTR3 is a cilia-localized GPCR that is also expressed in the PVN (32), including a subset of MRAP2-expressing neurons (Supplemental Figure 7, D and E). Interestingly, the absence of MRAP2 did not affect ciliary localization of SSTR3 (Supplemental Figure 8).

## Discussion

Ablation of primary cilium by conditionally knocking out *Ift88* in adult mice leads to obesity — either when deleted ubiquitously, specifically in neurons, or only in the PVN — directly implicating PVN cilia in the control of feeding behavior (13, 19).

The composition of the primary ciliary membrane is different from that of the surrounding plasma membrane, as it is enriched for proteins involved in specific forms of signaling (33). We previously identified MC4R as one of a select subset of GPCRs that localizes to cilia. Moreover, antagonizing Gαs signaling specifically at the primary cilium of MC4R-expressing PVN neurons also leads to obesity (13), suggesting that not only does MC4R localize to cilia, but it functions in cilia to mediate energy homeostasis. Importantly, human obesity-associated *MC4R* mutations affecting its third intracellular loop (a domain previously implicated in the ciliary localization of other GPCRs; ref. 34) impair MC4R ciliary localization without affecting its trafficking to the cell membrane or its ability to couple to G proteins (12). In the present study, we demonstrate that a ciliary GPCR accessory protein, MRAP2, restrains feeding by acting in MC4R-expressing neurons to direct its associated GPCR, MC4R, to cilia. These studies provide multiple concordant lines of evidence demonstrating that ciliary localization is critical for MC4R function in humans and mice. Since MC4R and MRAP2 are essential for suppressing feeding behavior and since MC4R fails to localize to primary cilia in *Mrap2*^–/–^ mice, it is likely that the failure of MC4R to localize to cilia accounts for the obesity observed in MRAP2-deficient mice and humans.

Previous in vitro studies in unciliated cells have reported conflicting effects of MRAP2 on MC4R function: one study reported that MRAP2 does not affect MC4R activity (30), another suggested that MRAP2 may inhibit MC4R activity (24), and others reported that MRAP2 increases its response to ligand (23, 25). MRAP2 was also reported to either positively or negatively regulate MC4R activity depending on their relative concentrations (26). Similarly, MRAP2 has been reported to either decrease (24) or modestly increase (25) MC4R cell-surface expression. It will be of interest to repeat these assays in ciliated cells.

The ciliary targeting of class A GPCRs such as SSTR3, D1R, MCHR1, and HTR6 depends on interactors such as Tubby family members and the IFT-A complex (32, 35–37). In contrast, the dependence of MC4R on MRAP2 for ciliary trafficking reveals a more specific requirement. For example, we found that, although MRAP2 and SSTR3 colocalize at primary cilia of some PVN neurons, SSTR3 does not require MRAP2 for ciliary localization. Therefore, MRAP2 may be a ciliary trafficking chaperone for MC4R, rather than a component of the general ciliary trafficking machinery. Ciliary accessory proteins such as MRAP2 may represent a site of regulation for the ciliary localization and function of their associated receptors.

Although MRAP2 is not required generally for ciliary GPCR localization, it may associate with other GPCRs beyond MC4R, including GHSR1a (22) and PKR1 (21, 21). Indeed, association of MRAP2 with receptors other than MC4R could also explain the differences in weight phenotypes caused by removing *Mrap2* globally or specifically in MC4R-expressing neurons. Specifically, we found that inactivating *Mrap2* specifically in MC4R-expressing neurons causes, like *Mc4R* loss-of-function, early-onset hyperphagia and obesity, whereas germline inactivation of *Mrap2* caused a milder phenotype, with late-onset hyperphagia and obesity (23, 29). Therefore, whether MRAP2 also promotes the ciliary function of orexigenic receptors should be assessed.

The role of primary cilia in metabolism (12, 18, 38–40) has motivated screens in heterologous cell culture systems to identify ciliary GPCRs that control energy homeostasis. Previously described ciliary GPCRs include the melanin-concentrating hormone receptor 1 (MCHR1) (34) and the neuropeptide y receptor 2 (NPY2R) (41, 42), which have also been shown to be implicated in energy homeostasis. However, the necessity of an accessory protein like MRAP2 for ciliary trafficking of specific GPCRs was not considered, suggesting that these screens may have missed a number of ciliary GPCRs, including MC4R (41, 42). Whether other ciliary GPCRs use accessory proteins for ciliary trafficking is an open question but is hinted at by studies demonstrating that proteins, such as rhodopsin, localize robustly to cilia in their native cell types but less well in heterologous ciliated cells (43). Our findings, therefore, suggest that it will be essential to consider the role of accessory proteins in future work both trying to uncover new ciliary receptor and working with these receptors in vitro to get as close as possible to physiological conditions.

Our study reveals that MRAP2 is required for the localization of MC4R to the primary cilia and the function of MC4R neurons. Within the context of the emerging connection between primary cilia and energy homeostasis (12, 18, 38–40), the observations described here further suggests that all genes controlling localization of MC4R to primary cilia are candidate genes for human obesity.

## Methods

### Cell culture and transfections

#### Expression plasmids.

MC1R-GFP, MC2R-GFP, MC3R-GFP, and MC5R-GFP expression constructs were constructed as previously described for MC4R-GFP (44). Plasmids encoding MRAP1-FLAG and MRAP2-FLAG were obtained from Patricia M Hinkle (University of Rochester Medical Center, Rochester, New York, USA) (30).

#### Ciliary expression of MCRs and MRAPs in cultured cells.

All IMCD3 cells were generated from a parental mouse IMCD-Flpln line (Thermo Fisher Scientific). IMCD3 cells were transfected using X-tremeGENE 9 DNA Transfection Reagent (06365809001, Roche). The transfection reagent was diluted in OptiMEM (Invitrogen) and incubated at room temperature for 5 minutes. Then, the mixture was added to the diluted plasmids in a 6:1 ratio (6 μL transfection reagent to 1 μg DNA) and incubated at room temperature for 20 minutes. In total, 50,000 cells in suspension were added to the transfection mixture in a 24-well plate. Transfected cells were switched to starvation media after 24 hours and fixed 16 hours afterward. Double plasmid transfections were performed by diluting equal mass of each vector. Three independent transfections were carried out per condition.

#### Cell imaging.

In total, 50,000 cells were seeded for transfection on acid-washed 12 mm #1.5 cover glass (Fisherbrand, Thermo Fisher Scientific) in a 24-well plate. Starved cells were fixed in phosphate buffered saline (PBS) containing 4% paraformaldehyde (Electron Microscopy Sciences) for 15 minutes at room temperature and permeabilized in ice-cold 100% methanol (Thermo Fisher Scientific) for 5 minutes. Cells were then further permeabilized in PBS containing 0.1% Triton X-100 (BP151-500, Thermo Fisher Scientific), 5% normal donkey serum (017-000-121, Jackson ImmunoResearch), and 3% BSA (BP1605-100, Thermo Fisher Scientific) for 30 minutes. Permeabilized cells were incubated with specified antibodies (i.e., mouse IgG2b monoclonal anti–acetylated tubulin antibody [T6793, Sigma-Aldrich, 1:1,000] and mouse IgG1 monoclonal anti–FLAG M2 antibody [F1804, Sigma-Aldrich, 1:500]) for 1 hour, washed with PBS, and incubated with dye-coupled secondary antibodies (Jackson ImmunoResearch) for 30 minutes. Cells were then washed with PBS, stained with Hoechst DNA dye, and washed with PBS before mounting with Fluoromount G (Electron Microscopy Sciences).

The anti-MRAP2 antibody was validated in a stable clonal cell line expressing MC4R-3xNeonGreen and MRAP2-3xFlag. The 2 proteins are produced in stoichiometric amount as they are encoded within a single mRNA and separated by a self-cleaving T2A peptide (pEFα^ΔTATA^:MRAP2-3xFLAG-T2A-IgK-HA-Halo-MC4R-3xmNeonGreen). The parental line, IMCD3 FlpIn, was used as negative control. Both cell lines were stained as mentioned above with rabbit anti-MRAP2 (17259-1-AP, Proteintech, 1:50) and mouse anti–acetylated tubulin (6-11-B, T6793, Sigma, 1:1,000). MC4R-3xNeonGreen was detected by endogenous fluorescence.

Cells were imaged in a widefield fluorescence DeltaVision microscope (Applied Precision) equipped with a PlanApo 60***×***/1.40NA objective lens (Olympus), a pco.edge 4.2 sCMOS camera, a solid state illumination module (Insight), and a Quad polycroic (Chroma). *Z* stacks with 0.2 μm separation between planes were acquired using SoftWoRx. The illumination settings were: 140 μW 390 nm wavelength for 0.15 seconds to image Hoechst, 222 μW 475 nm wavelength for 0.3 seconds to image Alexa Fluor 488–stained MCRs, 123 μW 543 nm wavelength for 0.3 seconds to image Cy3-stained acetylated tubulin, and 115 μW 632 nm wavelength for 0.15 seconds to image Cy5-stained FLAG-labeled MRAPs. Images were flat field corrected, background subtracted, and maximally projected using Fiji. Ciliary intensity measurements were also taken in Fiji as previously published (45). To compensate for the cell-to-cell differences in expression driven by the transient nature of the transfection, we normalized the ciliary intensity to the intensity at the cytoplasm using an ROI of equal area to that of the cilium.

### In vivo experiments

#### Animals.

Mice were housed in a barrier facility and maintained on a 12:12 light cycle (from 7 a.m. to 7 p.m.) at an ambient temperature of 23°C ± 2°C and relative humidity 50%–70%. Mice were fed with rodent diet 5058 (Lab Diet) and group housed up to 5. Experiments were performed with weight-matched littermates.

#### Descriptions on mouse lines used.

The *Mc4r^tm1(egfp)Vai^* mice have an EGFP tag inserted in frame at the C-terminus of the endogenous *Mc4r* locus (“*Mc4r^gfp^*”; ref. 12). The Mc4r^tm2(t2a Cre)Vai^ mice have a -t2a-Cre sequence inserted in frame at the C-terminus of the endogenous Mc4r locus (MC4R^t2aCre^; ref.^13^). Mice carrying a Flip recombinase (Tg[ACTFLPe]9205Dym) express a FLP1 recombinase gene under the direction of the human ACTB promoter (The Jackson Laboratory). *Arl13B-GFP^tg^* transgenic mice ubiquitously expresses a GFP-tagged version of the ciliary protein ARL13B, under the control of the CAG promoter (Markus Delling, UCSF, San Francisco; ref. 31).

#### EUCOMM MRAP2 mice.

EUCOMM *Mrap2*-KO–first allele (“tm1a”) mice carry an *frt*-flanked β-gal gene and neo cassette preventing widespread expression of the *Mrap2* gene (EUCOMM tm1a allele or *Mrap2^–/–^,* C57BL/6-*Mrap2^tm1a(EUCOMM)Wtsi^*/Jsbgj, The Jackson Laboratory). When mice harboring this allele are crossed into an actin-FLpE background, the frt-flanked cassette is excised and MRAP2 WT function is restored (EUCOMM tm1c allele or *Mrap2^fl/fl^*). After Flip-mediated excision, a loxP-flanked Exon 4 remains, which allows for MC4R cell-specific deletion when crossed to *Mc4r*-*t2a-Cre–*knock-in mice (*Mc4r^t2aCre/t2aCre^ Mrap2^tm1c/tmc1^* or *Mc4r^t2aCre/t2aCre^ Mrap2^fl/fl^*). We obtained *Mc4r^t2aCre/t2aCre^ Mrap2^fl/fl^* and *Mc4r^t2aCre/t2aCre^ Mrap2^+/+^* littermates by crossing *Mc4r^t2aCre/t2aCre^ Mrap2^fll+^* mice. All mice were maintained on a mixed background.

### Stereotaxic AAV-injection surgeries

Eight-week-old *Mrap2^fl/fl^* *Mc4r^gfp^* females mice (*n* = 4) were injected unilaterally with pAAV-Ef1a-mCherry- IRES-CRE (Addgene, 55632-AAV8; http://n2t.net/addgene:55632; RRID:Addgene_55632). Animals were anesthetized with an initial flow of 4% isoflurane (Dechra), maintained under anesthesia using 2% isoflurane, and kept at 30°C–37°C using a custom heating pad. The surgery was performed using aseptic and stereotaxic techniques. Briefly, the animals were put into a stereotaxic frame (KOPF Model 1900), the scalp was opened, the planarity of the skull was adjusted, and a hole was drilled (PVN coordinates: AP = –0.8, ML = –0.2, DV = –5.3). A volume of 300 nL was injected at a rate of 0.1 μL/min. Animals were given preoperative analgesic (buprenorphine, 0.3 mg/kg, Covetrus) and postoperative antiinflammatory meloxicam (5 mg/Kg, Pivetal) and were allowed to recover at least 10 days, during which time they were single housed and handled frequently. The mice were calorie restricted at 75% for 2 weeks prior to perfusion.

### Brain imaging

Mice were perfused transcardially with PBS, followed by 4% paraformaldehyde fixation solution. Brains were dissected and postfixed in fixation solution at 4°C overnight, soaked in 30% sucrose solution overnight, embedded in OCT (Tissue-Tek), frozen, and cut into 20–35 μm coronal sections before being stored at –80°C until staining.

After washing, sections were blocked for 1 hour in 50% serum (goat, MilliporeSigma, S26; donkey, MilliporeSigma, D9663), 50% antibody buffer (1.125% NaCl [MilliporeSigma, S5886], 0.75% Tris base (Thermo Fisher Scientific, BP152), 1% BSA (Thermo Fisher Scientific, BP1600), 1.8 % L-Lysine (Alfa Aesar, J62225), and 0.04% sodium azide (Millipore Sigma), followed by incubation with primary antibody overnight at 4°C (chicken anti-GFP, Abcam, ab13970, 1:250; rabbit anti-Adcy3, Santa Cruz Biotechnology, sc-588, 1:500; rabbit anti-MRAP2, Proteintech, 17259-1-AP, 1:200; and goat anti-SSTR3 [M-18], Santa Cruz Biotechnology, sc-11617, 1:200). After washing, sections were incubated with secondary antibodies for 1 hour at room temperature (goat anti–chicken Alexa Fluor 488 [Invitrogen, A11039]; goat anti–rabbit Alexa Fluor 633 [Invitrogen, A21070] or 555 [Invitrogen, A21429]; donkey anti–chicken Alexa Fluor 488 [Invitrogen, A78948]; donkey anti–rabbit Alexa Fluor 647 [Invitrogen, A-31573]; donkey anti–goat Alexa Fluor 555 [Invitrogen, A21432]; all 1:500), washed, and stained with Hoechst (1:5,000; Invitrogen, H3570). They were then washed and mounted with Prolong Diamond antifade Mountant (Invitrogen, P36970).

The anti-MRAP2 antibody was validated in vitro in transfected cells (Supplemental Figure 4A) and in vivo on brain sections from *Mrap2* WT mice compared with MRAP2-KO sections (Supplemental Figure 4B).

In Figure 5, the immunofluorescence stainings were performed on brain sections from mice that were calorie restricted for a week at 75% of baseline food intake on regular chow and fasted for 24 hours prior to perfusion. In Supplemental Figure 4 (validation of MRAP2 antibody), the staining for MRAP2 and GFP was performed on brain sections from mice expressing MC4R-GFP, either WT or KO for MRAP2 (MRAP2^1a/1a^). In Figures 3 and 4, and in Supplemental Figures 4 and 5, images were taken of brains from P6 pups, and from P8 pups in Supplemental Figures 6–8. The brain of 1 P8 mouse was assessed in Supplemental Figure 6. The sex of P6 and P8 mice could not be determined because the anogenital distance is hardly measurable at such a young postnatal age. Of note, direct colocalization of MRAP2 with ADCY3 could not be assessed in vivo, as the primary antibodies to detect both proteins are produced in rabbits. We therefore performed immunofluorescence staining on 2 separate sets of slides to demonstrate that MC4R colocalizes with ADCY3 and that MC4R then colocalizes with MRAP2. MRAP2 localization to primary cilia was also confirmed by colocalization with arl13-GFP (Figure 3, A–C).

### Microscopy

The images used for the assessment of MC4R-GFP at the cilium of MRAP2 WT versus KO were acquired with a Leica SP5 (*Z* stack, 0.5 μm steps). The images used to assess the colocalization of MC4R-GFP and MRAP2 in P6 mice were acquired with a Leica SP8 with resonant scanner. The SSTR3 imaging and MRAP2 immunofluorescence in MC4R-GFP adult mice were acquired with a Nikon W1 wide field-of-view spinning disk confocal with Andor Zyla sCMOS camera (*Z* stack, 0.26 μm steps). Images in Supplemental Figure 4 were acquired on a CSU-W1/SoRa spinning disk microscope, with dual camera Hamamatsu ORCA-FusionBT (*Z* stack, 0.2 μm steps).

### Image processing

Images were processed with Fiji. Maximal intensity *Z* projections are from at least 20 slices over 15–20 μm. Quantified slices were matched for the number of slices projected and settings.

### Quantification of ciliary localization in cultured cells and hypothalamus sections

Matched *Z* stack maximum projections were analyzed in Fiji. Relative ciliary enrichment was calculated as follows: each primary cilium was manually defined by a segmented line following ADCY3^+^ (in vivo) or ACTB^+^ (in vitro) signal. This 2D space was then used to the pixel intensity of the other channels (integrated density [IntDen]). Ciliary intensity of MC4R-GFP was then calculated as the IntDen of MC4R-GFP in the cilium, subtracting adjacent background (measured as IntDen of same defined area near the cilium). To calculate relative cilia enrichment, the IntDen (cilium) was divided by the IntDen (cell body), measured in the closest cell body (as defined by the presence of a Hoechst^+^ nucleus). Enrichment > 1, therefore, indicates higher localization of the receptor at the primary cilium compared with the cell body. Figure 2 details an in vitro experiment in which data are from 3 different replicates and are represented as a superplots, and *n* = 20–40 ciliated cells per condition were imaged and analyzed. Figure 4 indicates an in vivo experiment in which 60–90 cilia per mouse were quantified.

### Mouse metabolism studies

For experiments presented in Figure 1, mice were weaned at 4 weeks of age, were single housed, and their food intake was measured manually every 24 hours for 4 consecutive days and averaged. Food intake data were excluded if the mouse lost a significant amount of weight because of single-housing stress. The animals were then housed in groups of 5, and their weight was measured weekly until 12 weeks of age. Food intake was again assessed as described at 12 weeks of age. Body composition was assessed by EchoMRI at 4 and 12 weeks of age. EE was measured by Comprehensive Lab Animal Monitoring System (CLAMS) at 8 weeks of age (Columbus Instruments). Mice were tested over 96 continuous hours, and the data from the last 48 hours were analyzed. EE is expressed in terms of kcal per hour and was calculated using the Lusk equation: EE = (3.815 + 1.232 × respiratory exchange ratio) × VO_2_. It was analyzed with CalR app software (46). The interaction between the effect of body weight and genotype was quantified in a multivariate linear regression model adjusted for sex using the lm() function of R software (47).

### Data availability

The data that support the findings of this study are available from the corresponding author upon request.

### Statistics

Sample sizes were chosen based upon the estimated effect size drawn from previous publications and from the performed experiments. Data distributions were assumed to be normal, but this was not formally tested. All tests used are indicated in the figure legends. We analyzed all data using Prism 7.0 (GraphPad Software), except for EE data that was analyzed using the lm() function of R software (47). *P* ≤ 0.05 was considered significant..

### Study approval

All animal procedures were approved by the IACUC of the UCSF.

## Author contributions

A Bernard, JFR, MVN, and CV designed the research study and wrote the manuscript. A Bernard, ION, and XY conducted experiments and acquired and analyzed data. JC, EM, FBG, A Blake, KK, and SZ conducted experiments. FM analyzed data.

## Supplementary Material

Supplemental data

## Version 1 01/24/2023

Electronic publication
