# Supplementary material for: MRAP2 regulates energy homeostasis by promoting primary cilia localization of MC4R
Source: JCI Insight. 2023 Jan 24;8(2):e155900. doi: 10.1172/jci.insight.155900 (PMC9977312; doi:10.1172/jci.insight.155900)
Supplement: Supplemental data [file jciinsight-8-155900-s144.pdf]

SUPPLEMENTAL DATA

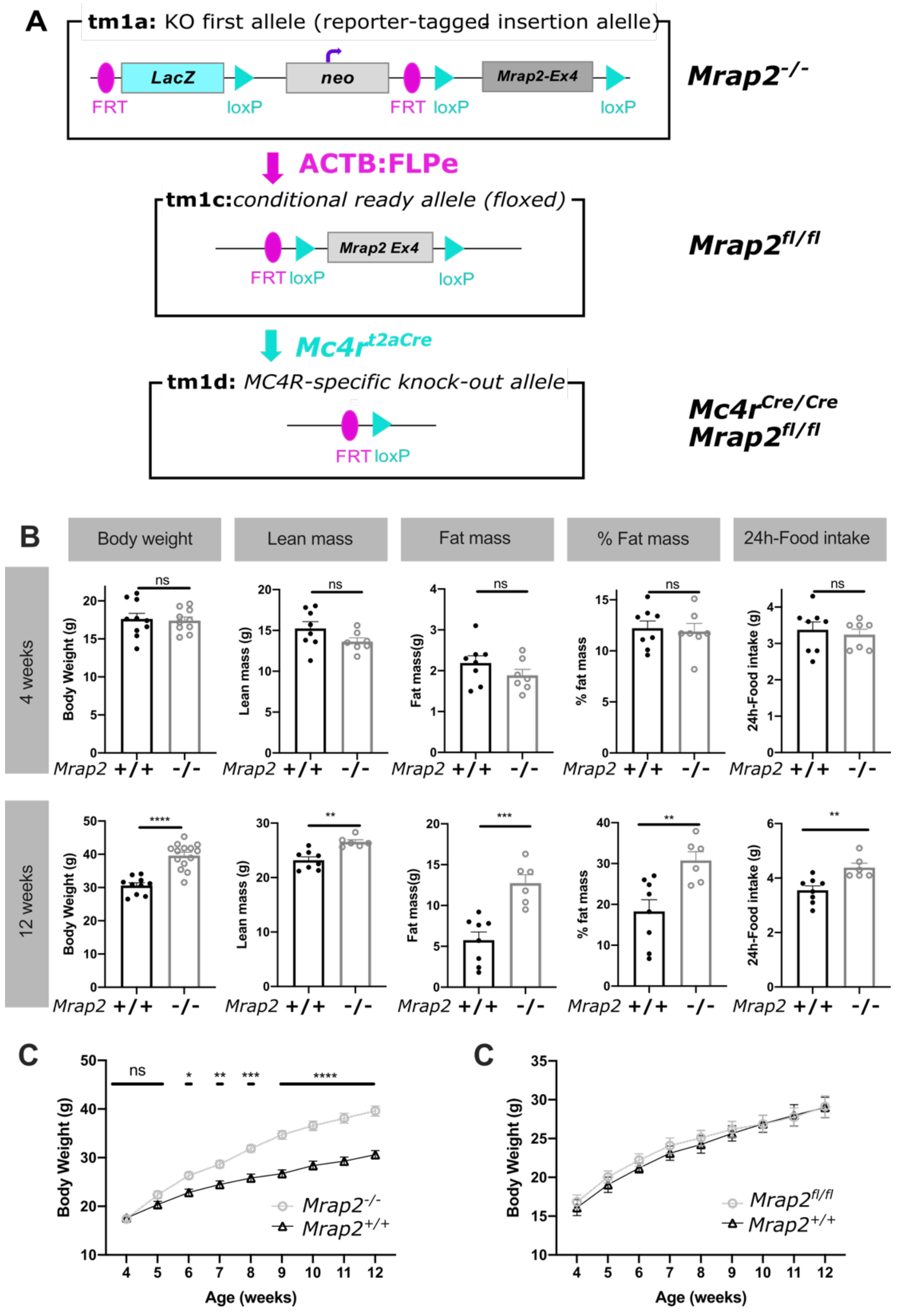

Figure S1: Phenotypical characterization of mice bearing EUCOMM MRAP2 tm1a and tm1c alleles. ***a*** EUCOMM allele nomenclature and specific crosses. EUCOMM *Mrap2* knockout-First allele ("tm1a") mice carry an *frt*-flanked β-gal gene and neo cassette preventing widespread expression of the *Mrap2* gene (EUCOMM tm1a allele or *Mrap2*$^{-/-}$). When mice harboring this allele are crossed into an actin-flip background (ACTB:FLPe), the frt-flanked cassette is excised and *Mrap2* wild-type function is restored (EUCOMM tm1c allele or *Mrap2*$^{fl/fl}$). After Flip-mediated excision, a loxP-flanked Exon 4 remains, which allows for *Mc4r* cell-specific deletion when crossed to *Mc4r-t2a-CRE* knock-in mice (*Mc4r*$^{t2aCre/t2aCre}$ *Mrap2*$^{fl/fl}$). ***b*** Body composition and 24h-food intake at 4 and 12 weeks of age (top and bottom panel respectively) of male mice homozygous for the EUCOMM *Mrap2*^{tm1a} allele (*Mrap2* whole body knockout, *Mrap2*$^{-/-}$, n=14) compared to their wildtype littermates (*Mrap2*$^{+/+}$, n=10). ***c*** Body weight curve of *Mrap2*$^{-/-}$ (n=14) compared to wildtype *Mrap2*$^{+/+}$ littermates (n=10). ***d*** Body weight curve of male mice homozygous for the EUCOMM *Mrap2*^{tm1c} allele (*Mrap2* floxed allele, *Mrap2*$^{fl/fl}$, n=7), compared to their wildtype littermates (*Mrap2*$^{+/+}$, n=10). Data are represented as mean ± SEM, *p<0.05, **p<0.01, ***p<0.001, ****p<0.0001, Student's unpaired t-test (column analysis); Mixed-effects model (REML) and Sidak's multiple comparisons tests (weight curves).

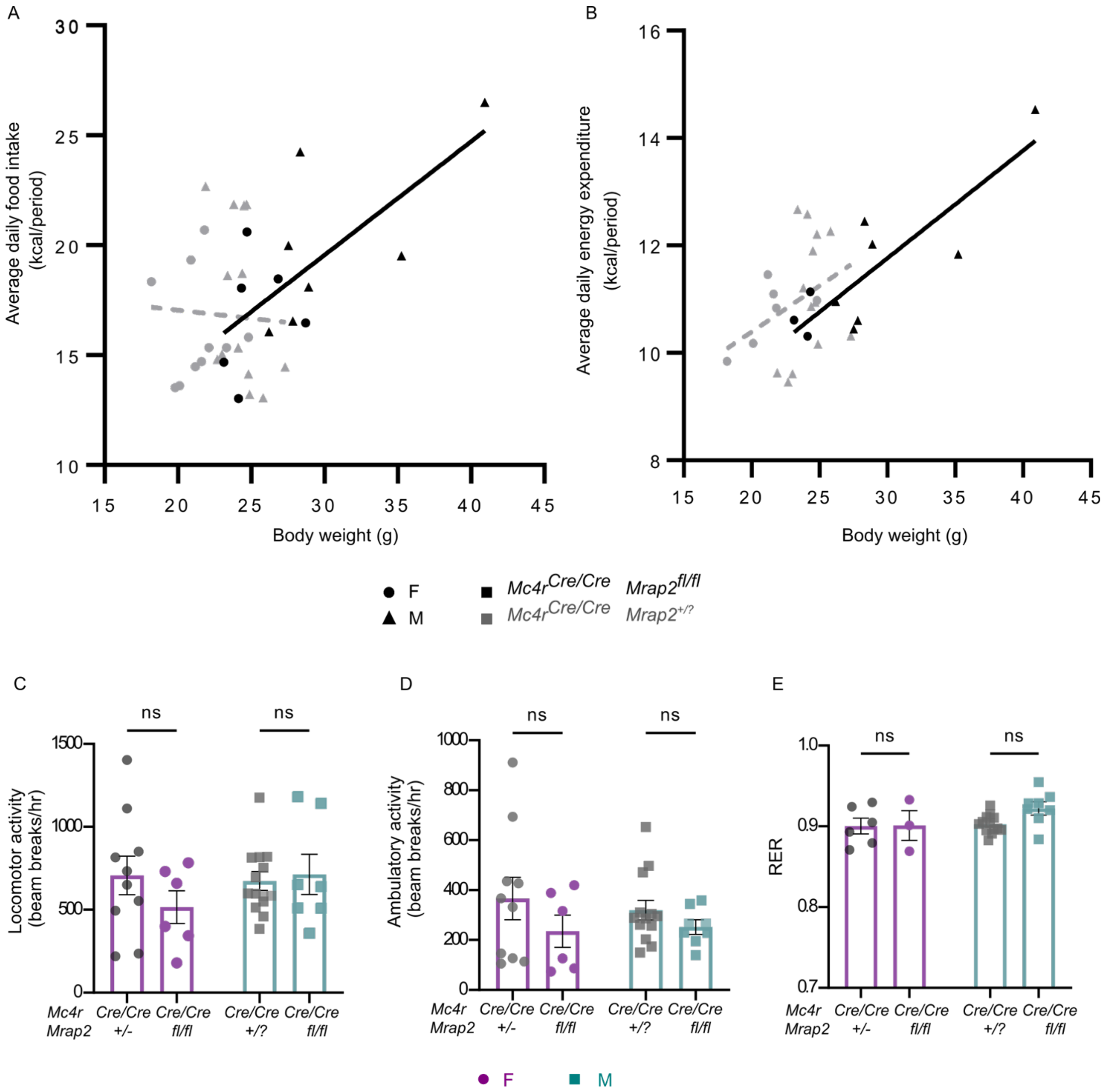

Figure S2: **Deleting MRAP2 in MC4R-expressing cells leads to an impairment in food intake regulation but not in energy expenditure or activity.** Average daily food intake (***a***, positive interaction between the effect of body weight and genotype, pValue*=0.049) and average daily energy expenditure (***b,*** no interaction between the effect of body weight and genotype, pValue=0.987) of $Mc4r^{t2aCre/t2aCre}$ $Mrap2^{fl/fl}$ vs $Mc4r^{t2aCre/t2aCre}$ $Mrap2^{+/?}$ male and female Mice measured by CLAMS.

The interaction between the effect of body weight and genotype was quantified in a multivariate linear regression model adjusted for sex. Locomotor activity (***c***), Ambulatory activity (***d***) and RER (***e***) were analyzed by two-way ANOVA with Sidak's multiple comparisons tests. ***a, c, d:*** $Mc4r^{t2aCre/t2aCre}$ $Mrap^{+/?}$ (n= 10 females, 13males); $Mc4r^{t2aCre/t2aCre}$ $Mrap2^{fl/fl}$ (n=6 females, 7 males); ***b, e:*** $Mc4r^{t2aCre/t2aCre}$ $Mrap^{+/?}$ (n= 6 females, 13males); $Mc4r^{t2aCre/t2aCre}$ $Mrap2^{fl/fl}$ (n=3 females, 7 males), 8 weeks old of age. Data represent the mean ± SEM.

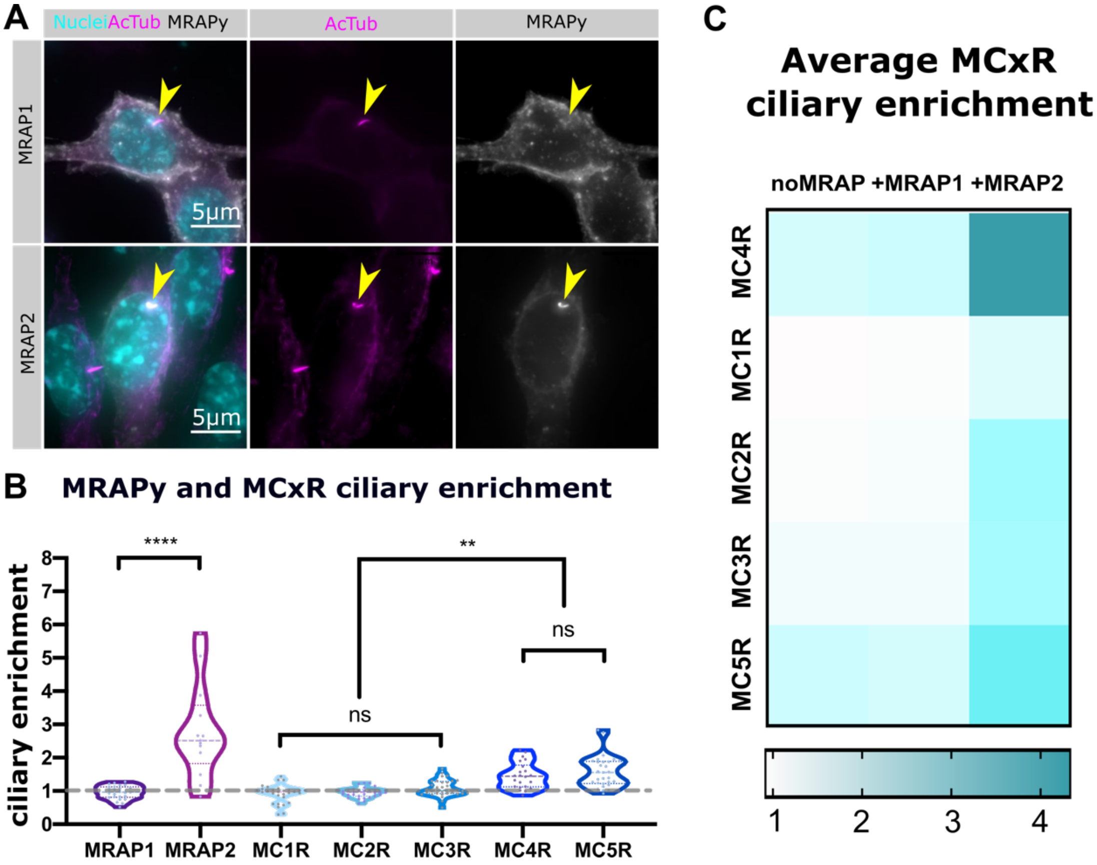

Figure S3: Ciliary enrichment of Melanocortin receptors, MRAP1 and MRAP2. ***a*** MRAP2 (lower panel) is enriched at the primary cilium when transfected in IMCD3 cells without any Melanocortin Receptor, while MRAP1 (upper panel) does not. Yellow arrows point at primary cilium of transfected cells. Scale bar 5 µm. ***b*** Quantification of the enrichment at the primary cilia of IMCD3 cells transfected with MRAPs and MCRs alone. ***c*** Heatmap displaying mean enrichment at the cilium when MCRs are transfected without MRAP (column 1), with MRAP1 (column 2) or MRAP2 (column 3). MRAP2 highly enriches MC4R localization at the cilium compared to other MCRs.

30-34 ciliated cells per condition were imaged and analyzed. Ciliary and cell body intensity of MCxR and MRAPy was measured with Fiji. Enrichment at the cilium is expressed as (integrated density at the cilium)/ (integrated density in the cell body). Enrichment >1 indicates higher localization of the protein at the cilium than at the cell body. Data are represented as violin plots, *p<0.05, ***p<0.001, ****p<0.0001, ordinary one-way ANOVA with Sidak's multiple comparisons test.

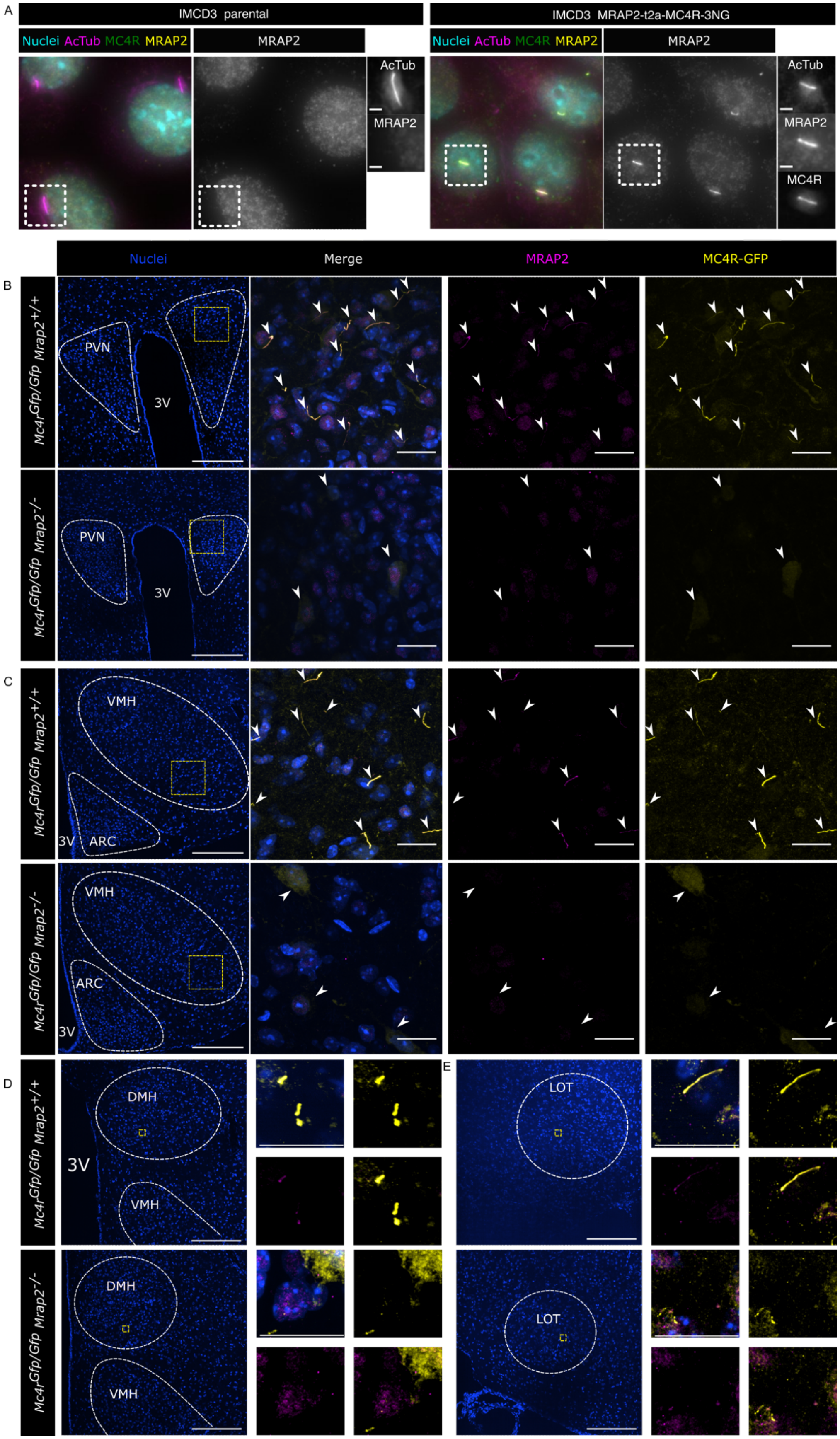
A
IMCD3 parental
Nuclei AcTub MC4R MRAP2
MRAP2
AcTub
MRAP2
IMCD3 MRAP2-t2a-MC4R-3NG
Nuclei AcTub MC4R MRAP2
MRAP2
AcTub
MRAP2
MC4R
Nuclei
Merge
MRAP2
MC4R-GFP
B
Mc4rGfp/Gfp Mrap2+/+
PVN
3V
Mc4rGfp/Gfp Mrap2-/-
PVN
3V
C
Mc4rGfp/Gfp Mrap2+/+
VMH
3V
ARC
Mc4rGfp/Gfp Mrap2-/-
VMH
ARC
3V
D
Mc4rGfp/Gfp Mrap2+/+
DMH
3V
VMH
Mc4rGfp/Gfp Mrap2-/-
DMH
VMH
E
LOT
LOT

Figure S4: Specificity of MRAP2 antibody.
a The anti-MRAP2 antibody detects a ciliary signal in IMCD3 cells co-expressing MRAP2 and MC4R-3NG (right). No ciliary signal is detected by the MRAP2 antibody in parental IMCD3 cells (left). MC4R-3NG was detected via the endogenous fluorescence of mNeonGreen (green), acetylated tubulin (AcTub, magenta) and MRAP2 (yellow) were detected via respective antibodies and DNA was stained with Hoechst (cyan). Grayscale images of each channel are shown in the inserts. Scale bar, 5 µm (main panels), 2µm (inserts). ***b-e*** No MRAP2 signal is observed in hypothalamic sections from *Mrap2* knockout animals. In wild-type sections ($Mc4r^{gfp}$ $Mrap2^{+/+}$), MRAP2 signal (magenta) can be found in cilia co-expressing MC4R (anti-GFP, yellow). In *Mrap2* knockout sections ($Mc4r^{gfp}$ $Mrap2^{-/-}$), no MRAP2 signal is detectable, including in the few cilia expressing MC4R. Nuclei stained with Hoechst (blue). Representative images from n=3 per group. Scale bar, left panels showing nuclei location 200µm; inserts, 20 µm.
PVN, paraventricular nucleus of the hypothalamus; VMH, ventromedial nucleus of the hypothamus; DMH, dorsomedial nucleus of the hypothalamus, ARC, arcuate nucleus; LOT, nucleus of the lateral olfactory tract; 3V, third ventricle.

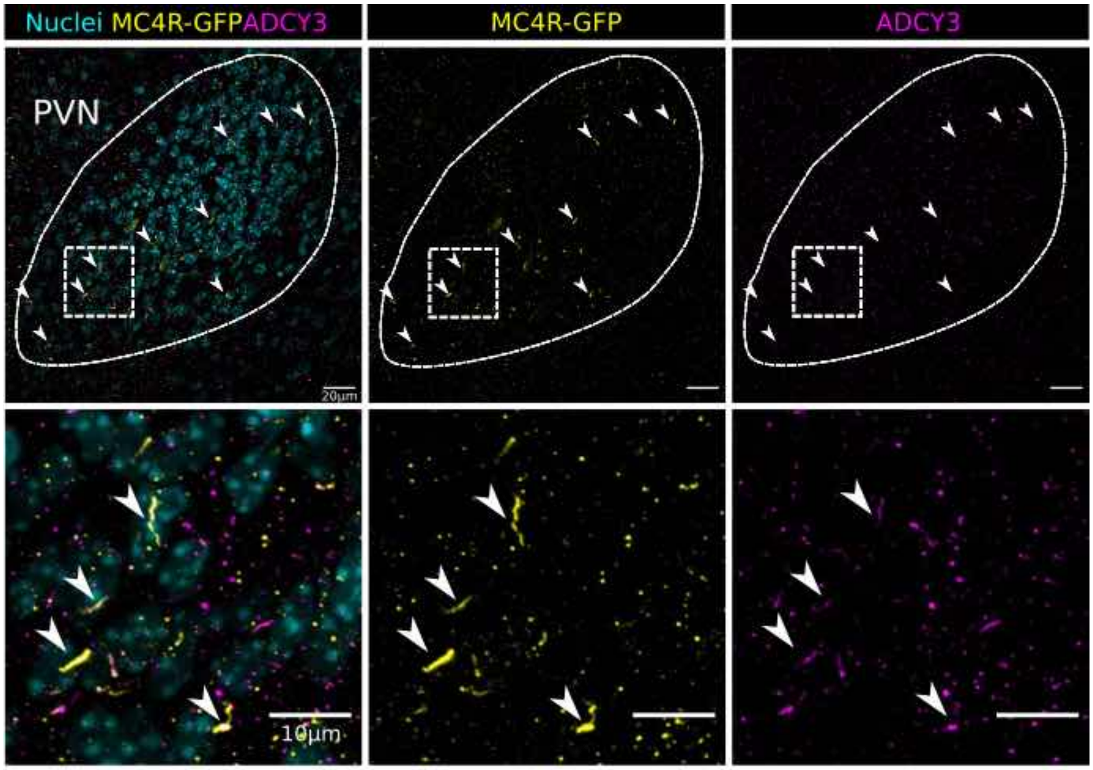

Figure S5: MC4R localizes at the primary cilium in P6 pups. MC4R-GFP (Yellow) colocalizes with the specific neuronal primary cilia marker ADCY3 (magenta) in the PVN of P6 pups. Scale bars: 20 μm (top) and 10 μm (bottom). Representative images of n=3, multiple bregmas were assessed per mouse.

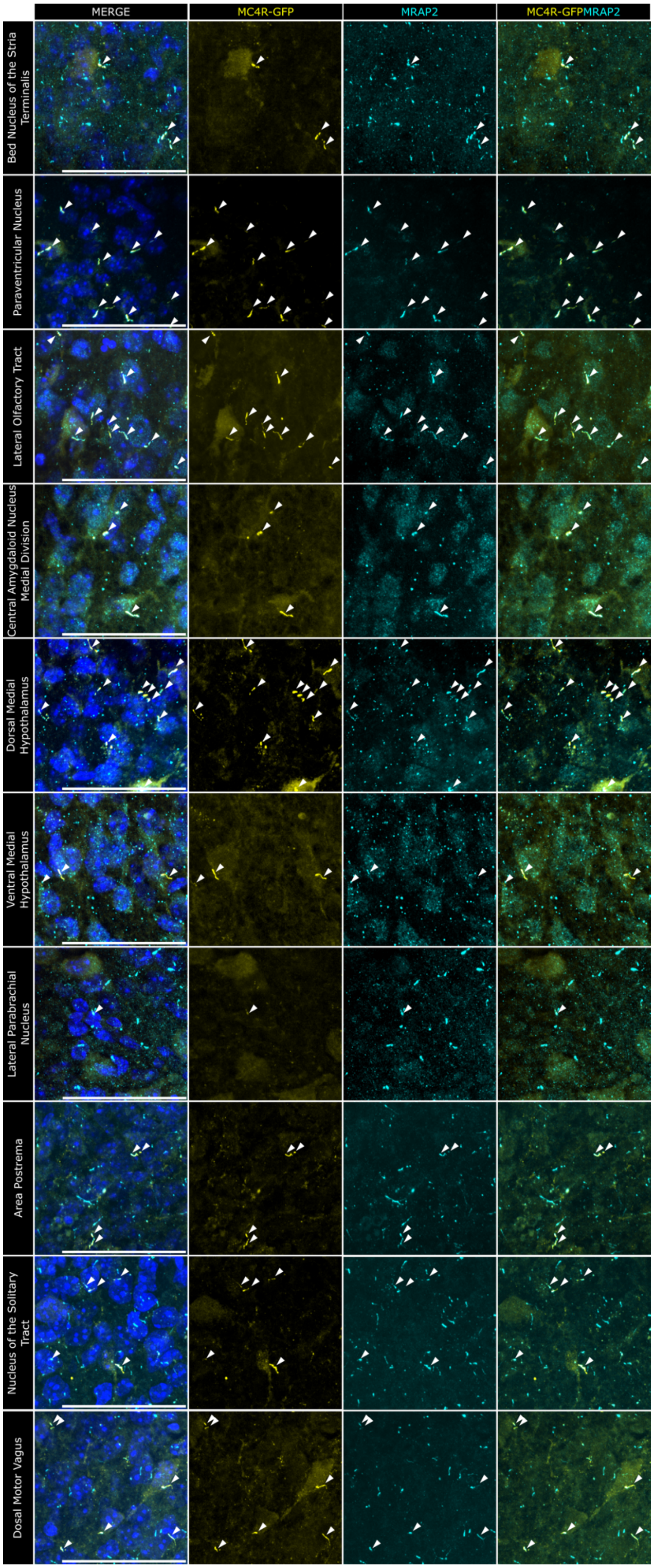
MERGE
MC4R-GFP
MRAP2
MC4R-GFPMRAP2
Bed Nucleus of the Stria Terminalis
Paraventricular Nucleus
Lateral Olfactory Tract
Central Amygdaloid Nucleus Medial Division
Dorsal Medial Hypothalamus
Ventral Medial Hypothalamus
Lateral Parabrachial Nucleus
Area Postrema
Nucleus of the Solitary Tract
Dosal Motor Vagus

Figure S6: Colocalization of MC4R and MRAP2 at the neuronal primary cilium in selected nuclei. Immunofluorescence imaging showing co-localization of MC4R-GFP (yellow) with MRAP2 (cyan) at the neuronal primary cilium. Nuclei are shown in blue. Primary cilium double positive for MC4R-GFP and MRAP2 are indicated by arrows. Scale bar, 50μm.

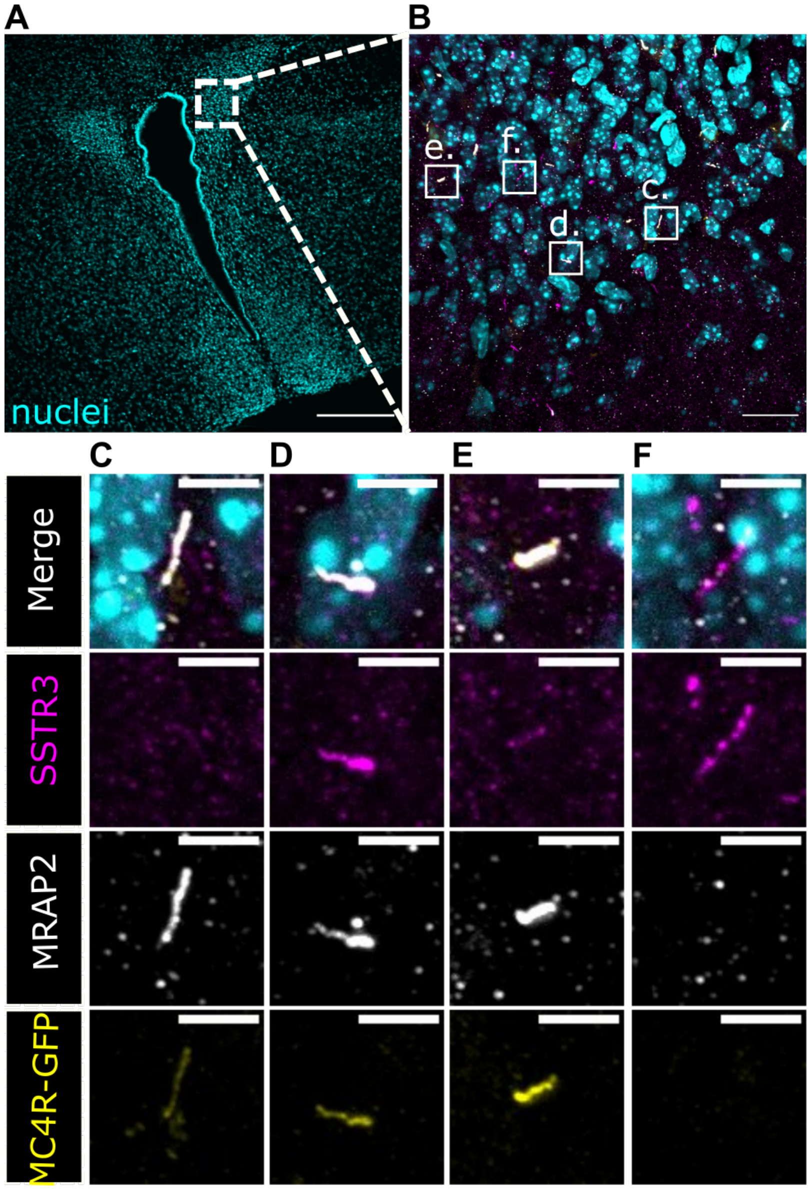

Figure S7: MRAP2 co-localizes with SSTR3 and MC4R in a subset of neurons in the PVN. ***a*** Low magnification image showing the position of the insert in b. Scale bar, 200 μm ***b*** Insert from *a*. Scale bar, 20 μm. ***c*** Primary cilium double positive for MC4R-GFP (yellow) and MRAP2 (white), but not SSTR3 (magenta). ***d*** and ***e*** Primary cilia triple positive for MC4R-GFP, MRAP2 and SSTR3. ***f*** Primary cilium positive for SSTR3 only. F-C scale bar, 5μm. Representative images, one of n=3.

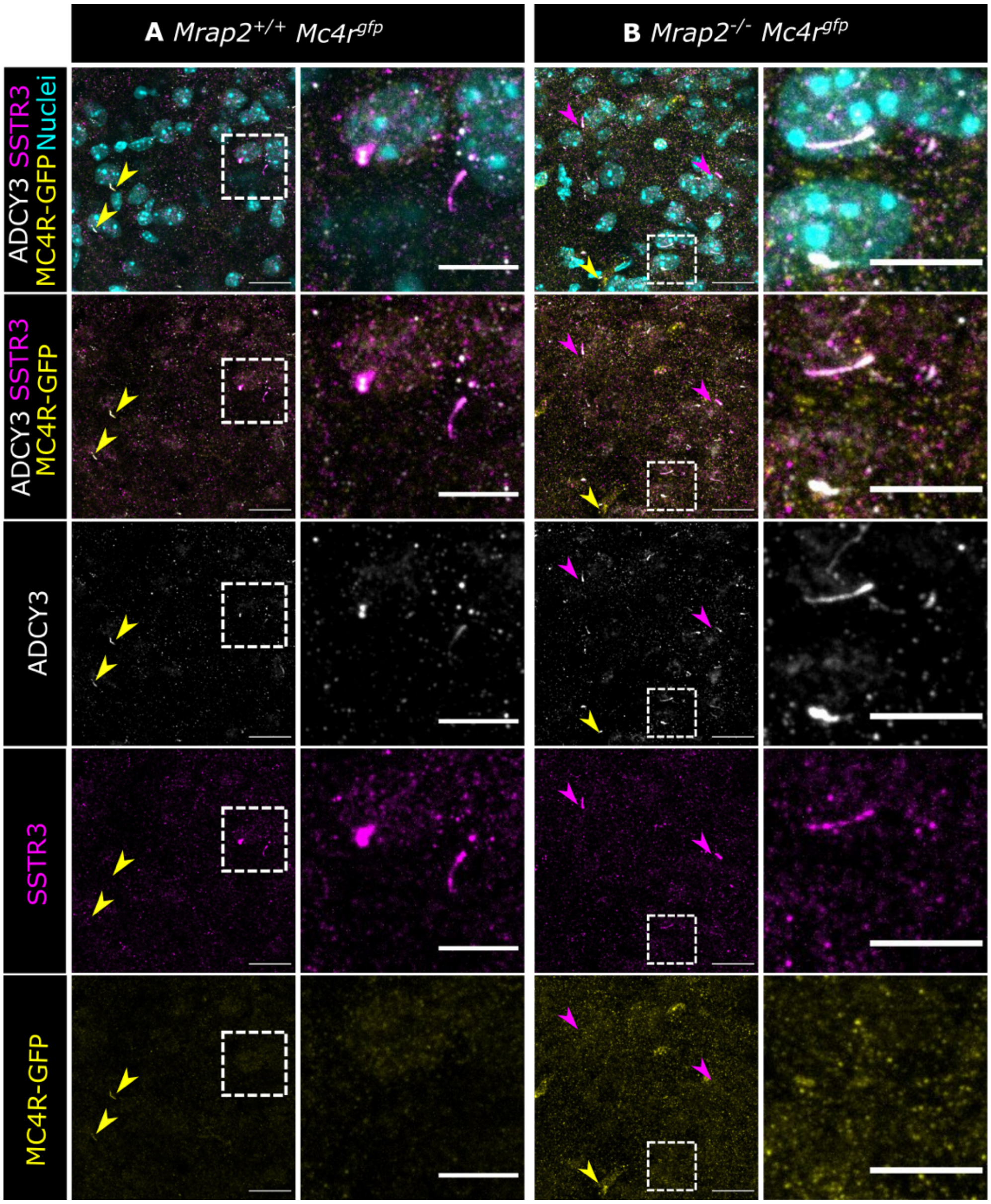

Figure S8: MRAP2 deletion does not impact SSTR3 ciliary localization. Immunofluorescence imaging of the PVN of a MRAP2 wild type (***a***) and a MRAP2 knockout mouse (***b***), showing co-localization of SSTR3 (magenta) with ADCY3 (white) at the neuronal primary cilium. Yellow arrows point at MC4R-GFP+ cilia, while magenta arrows point at SSTR3 positive cilia. Second and fourth columns are inserts of first and second columns showing SSTR3+ cilia in both MRAP2 wild type and mutant mice, respectively. Thin scale bar, 20μm; thick scale bar, 10μm. Representative images, one of n=3 per group.
